# Increasing surgical wait time does not increase the risk of node positive prostate cancer: Implications for surgical planning during the COVID-19 pandemic and beyond

**DOI:** 10.3389/fruro.2023.1132139

**Published:** 2023-03-09

**Authors:** Michael Zaliznyak, Rainey Horwitz, Facundo Davaro, Geoffrey H. Rosen, Katie S. Murray, Zachary Hamilton

**Affiliations:** ^1^ Department of Surgery, Saint Louis University School of Medicine, St. Louis, MO, United States; ^2^ Division of Urology, Saint Louis University School of Medicine, St. Louis, MO, United States; ^3^ Division of Urology, University of Missouri, Columbia, MO, United States

**Keywords:** prostate cancer, node positive, surgical wait time, COVID-19, prostatectomy

## Abstract

**Purpose:**

Prostate cancer (PCa) is a heterogeneous diagnosis, with a significant latency between diagnosis and risk of cancer specific mortality. During the COVID-19 pandemic, the need to balance the risk of COVID-19 exposure and resource allocation resulted in delays in non-emergent surgeries. We sought to assess if delays in surgical wait time (SWT) result in an increased risk of disease progression in the setting of clinically node positive PCa.

**Materials and methods:**

The National Cancer Database was queried for patients with cT1-3N0-1M0 PCa who underwent radical prostatectomy with lymph node dissection from 2010 to 2016. Patients were grouped based on pathologic node status (pN0 versus pN1). Outcomes including clinical tumor characteristics, hospital readmissions, and survival was correlated with length of SWT prior to radical prostatectomy.

**Results:**

A total of 218 patients with pN0 PCa and 805 patients with pN1 PCa met our inclusion criteria and were included in this study. Hospital length of stay and 30-day readmissions were similar between pN0 and pN1 patients. No significant association was detected between increased SWT and pN1 status among our patient population. Sensitivity multivariable analyses including only patients with Gleason 7-10 and excluding those who received androgen deprivation therapy prior to surgery showed similar findings that SWT was not associated with pN1 disease. With short term follow up, Kaplan-Meier analysis showed no significant difference in overall survival when stratified by SWT at 30-, 60-, 90-, or >90-day intervals.

**Conclusion:**

With the impact of the recent pandemic on healthcare and hospital systems, it is important to understand the effect that likely delays in SWT can have on patient outcomes. The findings described in this study suggest that delays in SWT may not result in adverse nodal disease progression among patients diagnosed with pathological node positive PCa. These results will be important to share with patients and their families when discussing treatment options and can result in improved patient outcomes and satisfaction with treatment regimens.

## Introduction

Prostate cancer (PCa) is a heterogeneous diagnosis, usually with a significant latency between diagnosis and risk of cancer specific mortality. Those with clinically detectable lymph nodes on conventional imaging may be offered definitive therapy, including radical prostatectomy (RP) and pelvic lymph node dissection. More recently, a strategy of active surveillance and delays in definitive therapy has emerged as an effective treatment modality for the management of PCa (1). Several recent studies have assessed the impact of such delays in surgery for patients with both low-risk and high-risk PCa and demonstrated that delaying surgery is safe in selected patients, and thus RP should be considered as low priority compared to other emergent and cancer related surgeries (2–4).

The COVID-19 pandemic placed unprecedented stress on patients, providers, and our overall healthcare system. The need to balance the risk of COVID-19 exposure and resource allocation had resulted in a shift towards telemedicine (5) and delays in several non-emergent surgeries, including RP (6). The reduction in RP cases led to increased surgical wait times (SWT) for many patients with PCa patients (7). Even if safe, such delays in SWT can result in increased patient anxiety (8), and thus proper counseling surrounding the topic of surgical timing is critical for ensuring optimal patient outcomes, especially in the setting of potential metastatic or advancement of disease. Although existing studies have demonstrated that surgical delays do not negatively affect outcomes in those with localized PCa (2, 3, 9–11), the effects of such delays on patients with clinically node positive disease is less well described.

We aimed to assess whether increases in SWT result in an increased risk of pathologic node positive disease in the setting of clinically node positive PCa. Our study aims to expand upon existing literature by assessing the impact of delays in SWT on disease progression among node positive PCa patients using data derived from the Commission on Cancer’s National Cancer Data Base (NCDB) Participant User File, making it the largest assessment of node positive PCa outcomes to date. These results will be important in assisting physicians with providing proper patient counseling, managing expectations, and ensuring optimal patient outcomes when discussing treatment options for patients with node positive disease.

## Materials and methods

### Data source

Data for this analysis was derived from the Commission on Cancer’s NCDB Participant User File for men with cT1-3N0-1M0 PCa who underwent RP with lymph node dissection from 2010 to 2016. The NCDB is a national cancer outcomes dataset that includes input from over 1500 Commission on Cancer-accredited centers in the United States. These data include all cancer patients treated at participating Commission on Cancer-accredited institutions and are estimated to capture over 70% of new cancer cases in the United States (12). Standardized coding definitions are utilized, and the data are freely available to participating institutions after applications for projects are accepted by the NCDB. The data used in the study are derived from a de-identified NCDB file. The American College of Surgeons and the Commission on Cancer have not verified and are not responsible for the analytic or statistical methodology employed, or the conclusions drawn from these data by the investigator.

### Data collection

Patient demographic variables collected included: age, race, Charlson comorbidity index, income status, facility type, and insurance status. Disease and operative outcomes included PSA, Gleason score on biopsy, and clinical tumor stage, pre-operative androgen deprivation (ADT), days from diagnosis to RP, length of hospital stay after surgery, surgical margin status, length of follow-up, 30-day readmission, and 30- and 90-day mortality. Patients were grouped based on pathologic node status (pN0 versus pN1).

### Statistical analysis

Continuous variables were reported as means ± standard deviations (SD) and compared with two-tailed t-tests. Categorical variables were reported as number (percentage) and compared with the Chi-Square test. Using factors that were deemed clinically significant, we performed multivariable logistic regression to identify whether disease progression associated with SWT. Time to death at increasing SWT intervals was computed using survival regression. Differences were considered significant where p-values were < 0.05. All analysis was performed with SPSS Statistics v26 software by IBM Corporation (Armonk, New York, United States).

## Results

### Study population

From a review of the Commission on Cancer’s NCDB Participant User File we identified a total of 1,491,140 adults diagnosed with PCa between 2010 and 2016. Of these, 1,481,554 patients were excluded for having obsolete Gleason data (given the change in NCDB coding in 2010). The remaining 9,586 were diagnosed with cT1-3N0-1M0, of which 1,869 underwent prostatectomy with lymph node dissection. Patients with missing Gleason scores (N=733), missing PSA value (N=186), unsampled lymph nodes (N=325), or missing data on surgical waiting time (N=56) were further excluded.

**Figure 1:**
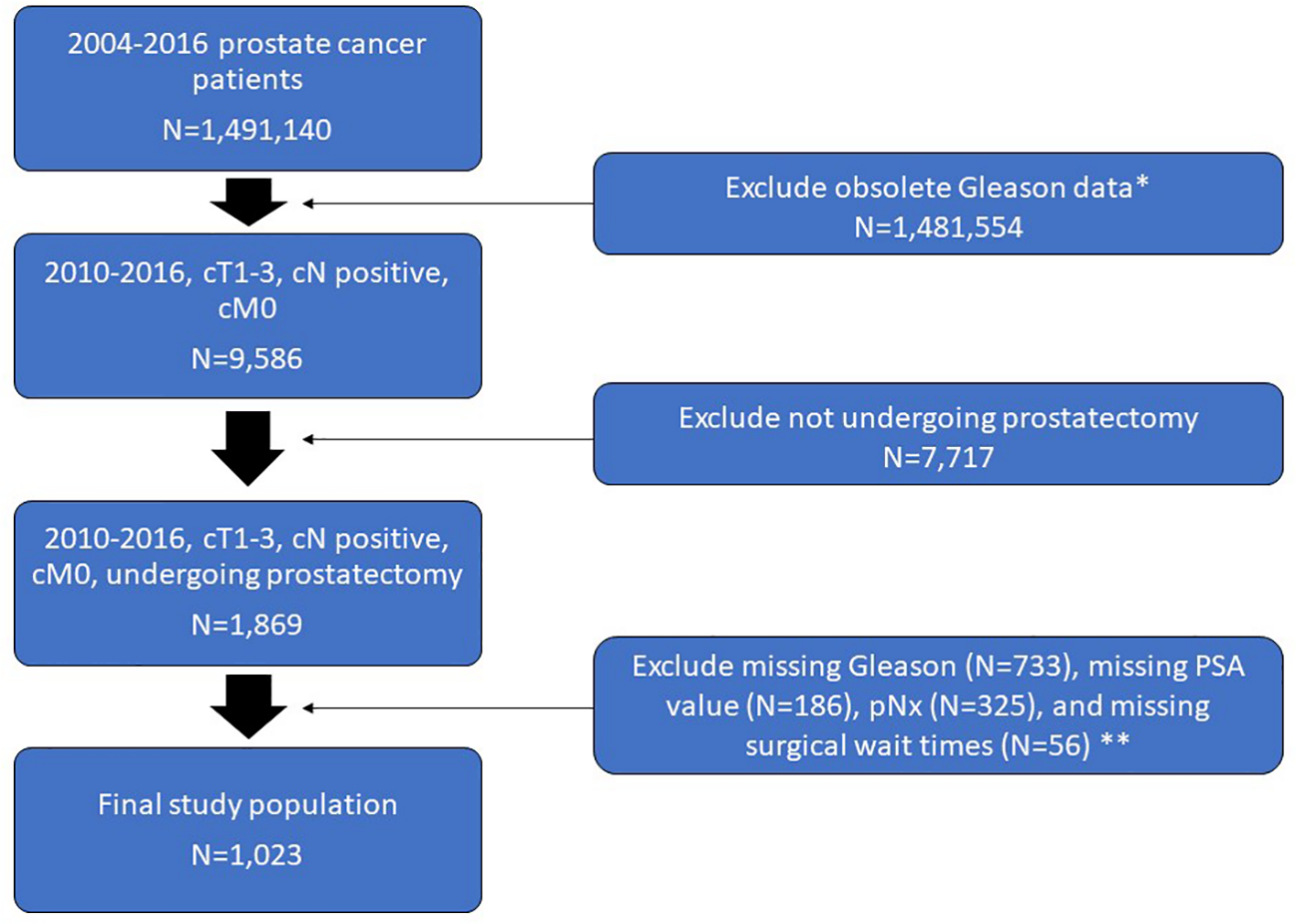
Flow chart of the study population selection including exclusion criteria. *Change in NCDB coding in 2010. **Overlap exists between patients who met one or more of these exclusion criteria.

A total of 1,023 patients (mean age ± SD, 61.7 ± 7.3 years) met our inclusion criteria and were included in our study population (**Figure 1**). Patients were stratified based on pathologic node status, Gleason score, PSA value, and clinical staging. Demographics of our study population are shown in **Table 1**.

**Table 1:** Patient demographics and clinical tumor characteristics.

Variable	All (n=1023)	pN0 (n=218)	pN1 (n=805)	p-value
**Mean Age**	61.7 ± 7.3	62.0 ± 7.0	61.6 ± 7.4	0.535
**Race**				0.480
** White**	871 (85.1%)	185 (84.9%)	686 (85.2%)	
** Black**	102 (10.0%)	25 (11.5%)	77 (9.6%)	
** Other**	50 (4.9%)	8 (3.7%)	42 (5.2%)	
**Charlson**				0.416
** 0**	836 (81.7%)	175 (80.3%)	661 (82.1%)	
** 1**	154 (15.1%)	36 (16.5%)	118 (14.7%)	
** 2**	26 (2.5%)	7 (3.2%)	19 (2.4%)	
** 3+**	7 (0.7%)	0 (0%)	7 (9.0%)	
**Income Status**				0.315
** <$38,000**	148 (14.5%)	38 (17.4%)	110 (13.7%)	
** $38,000-47,999**	189 (18.5%)	45 (20.6%)	144 (17.9%)	
** $48,000-62,999**	245 (23.0%)	50 (22.9%)	195 (24.3%)	
** $63,000+**	440 (43.1%)	85 (39.0%)	355 (44.2%)	
**High Volume Facility**	890 (87.0%)	195 (89.4%)	695 (86.3%)	0.257
**Insurance Status**				0.144
** Uninsured**	27 (2.6%)	2 (0.9%)	25 (3.1%)	
** Private Insurance**	611 (59.7%)	127 (58.3%)	484 (60.1%)	
** Medicaid**	23 (2.2%)	6 (2.8%)	17 (2.1%)	
** Medicare**	329 (32.2%)	80 (36.7%)	249 (30.9%)	
** Other Govt**	11 (1.1%)	1 (0.5%)	10 (1.2%)	
** Unknown**	22 (2.2%)	2 (0.9%)	20 (2.5%)	
**ADT Before Surgery**	212 (20.7%)	53 (24.3%)	159 (19.8%)	0.157

#### Clinical characteristics

The distribution of tumor characteristics was different between pN0 and pN1 patients, with pN1 patients having higher PSA values (p<0.001), higher Gleason scores (p<0.001), and higher tumor stage (p<0.001). Additionally, compared with pN0 patients, pN1 patients experienced significantly shorter waiting times between their initial diagnosis and time to RP(p=0.009) (**Table 2**).

**Table 2:** Clinical tumor characteristics.

Variable	All (n=1023)	pN0 (n=218)	pN1 (n=805)	p-value
**PSA**				**<0.001**
** <10**	268 (26.2%)	65 (29.8%)	203 (25.2%)	
** 10-20**	320 (31.3%)	44 (20.2%)	276 (34.3%)	
** >20**	435 (42.5%)	109 (50.0%)	326 (40.5%)	
**Gleason on Biopsy**				**<0.001**
** 6**	38 (3.7%)	24 (11.0%)	14 (1.7%)	
** 7**	296 (28.9%)	72 (33.0%)	224 (27.8%)	
** 8**	254 (24.8%)	51 (23.4%)	203 (25.2%)	
** 9**	395 (38.6%)	58 (26.6%)	337 (41.9%)	
** 10**	40 (3.9%)	13 (6.0%)	27 (3.4%)	
**cT Stage**				**<0.001**
** cT1**	281 (27.5%)	94 (43.1%)	187 (23.2%)	
** cT2**	291 (28.4%)	64 (29.4%)	227 (28.2%)	
** cT3**	451 (44.1%)	60 (27.5%)	391 (48.6%)	
**Days from dx to def treatment**				**0.009**
** <30 days**	50 (4.9%)	6 (2.8%)	44 (5.5%)	
** 31-60 days**	349 (34.1%)	67 (30.7%)	282 (35.0%)	
** 61-90 days**	302 (29.5%)	57 (26.1%)	245 (30.4%)	
** >90 days**	322 (31.5%)	88 (40.4%)	234 (29.1%)	

Bolded numbers are statistically significant, p<0.05.

#### Survival

**Figure 2:**
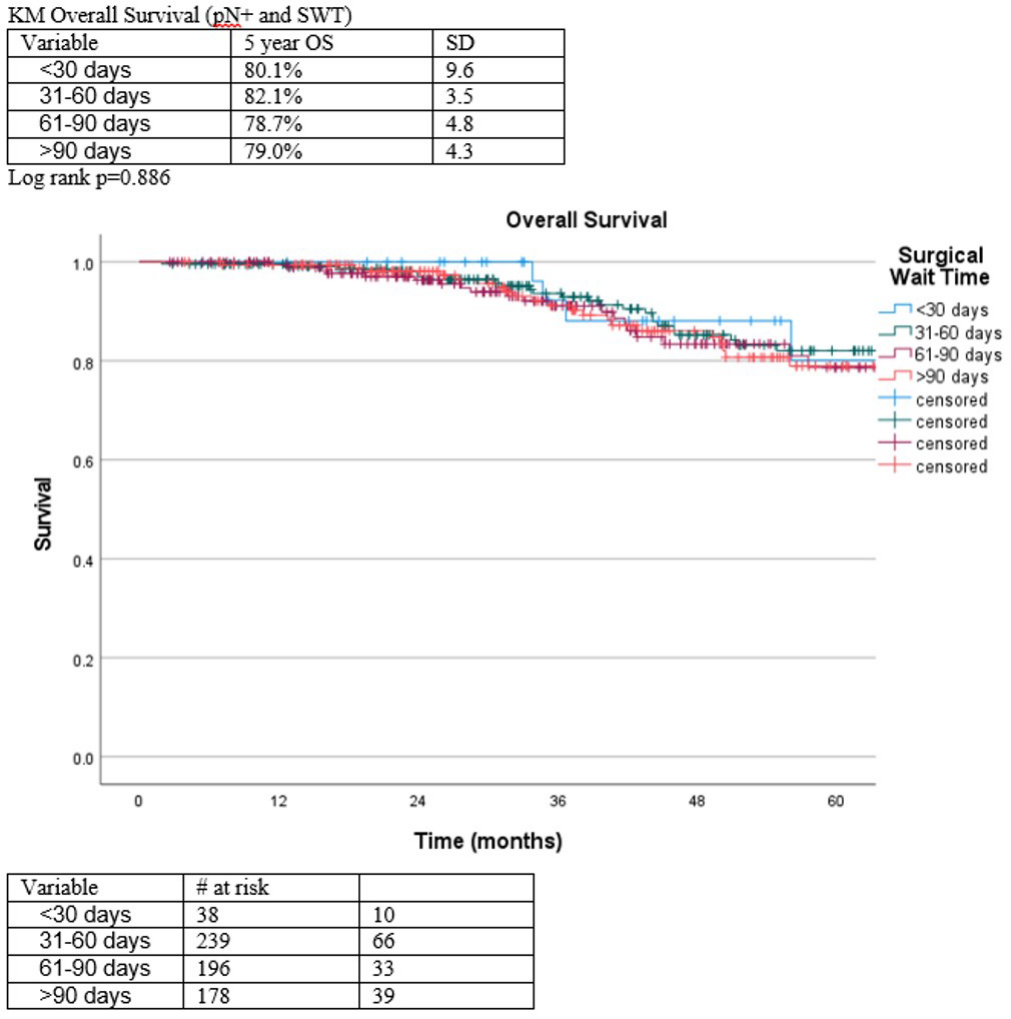
Estimated overall survival among pN+ patients with increasing surgical waiting times.

When stratifying pN+ patients by surgical wait time at 30-, 60-, 90-, or >90-day intervals, no significant difference in overall survival was detected on Kaplan-Meier survival analysis (**Figure 2**). Additionally, hospital length of stay and 30-day readmission rates were similar between pN0 and pN1 patients (**Table 3**). However, pN1 patients had significantly higher overall rates of mortality (9.7% vs. 4.1%, p=0.01) and shorter mean time to death (80.1 vs 85.0 months, p=0.013), when compared to pN0 patients (**Table 3**).

**Table 3:** Perioperative and survival outcomes.

Variable	All (n=1023)	pN0 (n=218)	pN1 (n=805)	p-value
**Days in Hospital**	2.1 ± 5.9	2.0 ± 3.5	2.1 ± 6.4	0.875
**Positive Margin**	510 (49.9%)	87 (39.9%)	423 (52.5%)	**0.001**
**30-day readmission**	22 (2.2%)	7 (3.2%)	15 (1.9%)	0.288
**Length of Follow Up (months)**	40.5 ± 22.4	40.7 ± 23.1	40.5 ± 22.2	0.935
**Mortality**	87 (8.5%)	9 (4.1%)	78 (9.7%)	**0.009**
** Within 30 Days of Treatment**	1 (0.1%)	0 (0%)	1 (0.1%)	1.000
** Within 90 Days of Treatment**	1 (0.1%)	0 (0%)	1 (0.1%)	1.000
** Mean Time to Death (months)**	81.5 ± 1.1	85.0 ± 1.7	80.1 ± 1.3	**0.013**

Bolded numbers are statistically significant, p<0.05.

#### Disease progression on multivariable logistic regression

Logistic regression was performed to evaluate for risk of pN+ disease after RP with lymph node dissection. We included age, race, Charlson score, use of androgen deprivation therapy, PSA, Gleason score on biopsy, clinical stage, and SWT in our model. Including all patients, we found that PSA >20, increasing Gleason score, and higher cT stage were associated with increased risk of pN+ disease; however, SWT did not demonstrate an association (p=0.129-0.348). A sensitivity analysis was performed only including patients with Gleason 8 or higher disease, and we found that PSA >20 and cT3 stage remained significantly associated with pN+ disease while SWT remained non-significant. An additional model that excluded patients who received ADT was performed and our findings were similar, as PSA >20, increasing Gleason score, and increasing clinical stage were significantly associated with increased risk of pN+ disease, but SWT had no association.

## Discussion

This study aimed to assess if increases in SWT resulted in an increased risk of pathologic node positive disease in the setting of RP for clinically node positive PCa. We were able to demonstrate that prolonged SWT was not associated with risk of node positive disease, and increases in SWT were not associated with reductions in overall patient survival. As expected, clinical characteristics such as increasing Gleason score, PSA >20, and increasing clinical stage portended node positive disease. These findings suggest that increasing SWT may not have direct effects on the risk of node positive PCa, and our findings provide a framework for patient counseling or preoperative risk stratification with surgical planning.

An understanding of the behavior of node positive PCa can assist in triaging patients as they await surgical treatments. This is especially relevant given the recent COVID-19 pandemic which has put a strain on global health care systems, resulting in delays in several non-emergent surgeries, including RP (2). Previous studies have described that patients who are subjected to delays in SWT may experience increased rates of depression and anxiety (13, 14), which can be magnified in the setting of a cancer diagnosis (15). Since the onset of the COVID-19 pandemic, the number of RP surgeries has fallen, resulting in significant treatment delay for many patients (7). Understanding the impact that these delays in treatment may impose on disease outcomes of node positive PCa can assist physicians with providing proper patient counseling, managing expectations, and ensuring optimal patient outcomes. It remains unclear the impact the pandemic has had on present and future surgical scheduling therefore, it is important for providers to reassure patients who may be concerned with their disease morbidity when facing delays in SWT. Our study population represents a timeframe prior to the COVD-19 pandemic, so the findings must be taken as hypothesis generating; however, these results can provide a framework for patient counseling and elective surgery triage. Although our findings largely support existing research describing the impact of delayed SWT on disease progression (3, 9, 10, 16, 17), we report several notable findings which warrant further consideration and our study is the first to highlight patients with clinically node positive disease.

We found that delayed SWT did not contribute to adverse nodal disease progression or diminished survival for patients with clinically node positive PCa. Our findings are in line with recent studies which have found that delayed RP did not adversely affect patients with low-risk (18), intermediate-risk (19), and high-risk PCa (3, 10, 19, 20). It has been well described that active surveillance is a safe alternative to immediate surgical treatment for PCa patients with low risk profiles (21, 22), however there is no consensus regarding the length of time that patients may delay definitive treatment, with higher risk disease (11). Our findings support that delaying surgery for up to 3 months is not associated with worse pathologic nodal outcomes; however, this must be carefully integrated with an understanding of the inherent selection bias of our analysis. In essence, surgeons may have unmeasured but individualized risk stratification that guides the timing of RP for clinically node positive patients, and our analysis suggests that surgeons can trust their judgement in this setting. Providers who may be considering delaying surgery for patients due to circumstance, as in the event of COVID-19, may be safely implemented for up to 3 months without worsening adverse events. These findings must be balanced with the provider’s own interpretation of metastatic potential and oncologic risk and patients’ comfort level with surgical and treatment timing.

A diagnosis of PCa may invoke fear and anxiety among patients (23), which may be exacerbated during periods of uncertainty such as in the context of an ongoing COVID-19 pandemic and the subsequent following era that may delay access to necessary treatments. As with any cancer diagnosis, PCa treatment plans should be prepared through a shared-decision making process between patients, their families, and their healthcare providers, taking into consideration several individualized patient factors. Our results are not meant to advocate for the intentional delay of treatment for patients diagnosed with PCa, however our findings support the conclusion that circumstantial or planned delays in SWT are not associated with overt adverse nodal disease progression or short-term morbidity. These findings may alleviate some of the stress and anxiety that patients may have and should be included in the body of evidence which is shared with patients and their families when discussing treatment strategies for PCa.

### Strengths and limitations

To our knowledge, this study is the largest assessment of the impact of SWT on disease progression and survival of patients diagnosed with clinically node positive PCa prior to RP. Additionally, our study stratifies patients by risk status which avoids allowing the results of low-risk PCa patients from underestimating the impact of delayed SWT on high-risk PCa patients. Limitations of our work include the potential for selection bias which is inherent to the retrospective nature of our data collection. Furthermore, the individualized patient risk factors or surgeon discretion that led to a delay in surgery or use of preoperative ADT is unknown within the dataset. From a surgical perspective, the extent and mapping of node dissections are unknown. It also not known whether or not reported patient mortality was related to PCa. Additionally, all data for this study was obtained from a review of the NCDB, which is a database composed of patients treated at Commission on Cancer accredited facilities and thus our results may not be representative of the overall population of patients who are not treated at such institutions.

## Conclusion

Our findings suggest that a delay in SWT does not result in impact on surgical or morbidity outcomes among patients diagnosed with pathological node positive PCa. The results described in this study will be important to share with patients and their families when discussing treatment options and can result in improved patient outcomes and satisfaction with treatment regimens.

## Data availability statement

The raw data supporting the conclusions of this article will be made available by the authors, without undue reservation.

## Ethics statement

Ethical review and approval was not required for the study on human participants in accordance with the local legislation and institutional requirements. Written informed consent for participation was not required for this study in accordance with the national legislation and the institutional requirements.

## Author contributions

FD, GR, KM, and ZH contributed to conception and design of the study. ZH performed the statistical analysis. MZ, RH, FD, and GR wrote sections of the manuscript. All authors contributed to the article and approved the submitted version.
